# The Annual costs of treating genital warts in the Public Healthcare Sector in Peru

**DOI:** 10.1186/s12913-021-07120-w

**Published:** 2021-10-14

**Authors:** Enrique M Saldarriaga, Cesar P. Cárcamo, Joseph B. Babigumira, Patricia J. García

**Affiliations:** 1 Epidemiology, STD and HIV Unit, School of Public Health and Administration, Universidad Peruana Cayetano Heredia, Lima, Peru; 2 The Comparative Health Outcomes, Policy, and Economics (CHOICE) Institute, University of Washington, Seattle, Washington USA; 3 Department of Global Health, School of Public Health, University of Washington, Seattle, Washington USA

**Keywords:** Genital warts, Cost-of-illness, Micro-costing, Peru

## Abstract

**Objectives:**

To estimate the cost of six different techniques used to treat Genital Warts and the annual average cost of treating a typical GW patient in Peru. To estimate the annual economic burden diagnosing and treating GW in the Peruvian public healthcare system.

**Methods:**

We developed a prevalence-based, cost-of-illness study from the provider’s perspective, the healthcare facilities under the purview of Peruvian Ministry of Health. We used an activity-based costing approach. We conducted primary data collection in three regions in Peru and supplemented it with governmental data. Uncertainty of the costing estimates was assessed via Monte Carlo simulations. We estimated the average cost and associated confidence intervals for six treatment options – three topical and three surgical – and the overall cost per patient.

**Results:**

The average treatment cost per patient was 59.9USD (95 %CI 45.5, 77.6). Given a population of 18.4 million adults between 18 and 60 years of age and a GW prevalence of 2.28 %, the annual cost of treating GW was 25.1 million USD (uncertainty interval 16.9, 36.6).

**Conclusions:**

This study provides the first quantification of the economic burden of treating genital warts in Peru and one of the few in Latin America. The costing data did not include other healthcare providers or out-of-pocket expenditures, and hence we present a conservative estimate of the COI of GW in Peru. Our findings bring attention to the financial burden of treating GW, a vaccine-preventable disease.

**Supplementary Information:**

The online version contains supplementary material available at 10.1186/s12913-021-07120-w.

## Introduction

Genital warts (GW) are the most common viral sexually transmitted infection (STI) globally [1]. They are manifestations of anogenital human papillomavirus (HPV). In particular, HPV-subtypes 6 and 11 are causative agents of the disease [2–4]. GW present as external skin lesions of the vulva, penis, anus and scrotum, and mucosal lesions of the vagina, cervix, and urethra [5].

Data related to the incidence and prevalence of GW varied significantly within countries [1]. To date, the only study that has estimated the prevalence of GW in Peru was conducted by García et al [6]. The authors conducted a survey among 100 physicians from public facilities to quantify the frequency of GW cases detected, as well as diagnosis practices and patients’ characteristics. The prevalence of GW among all Peruvian adults between 18 and 60 years of age was estimated in 2.28 % (95 % Confidence Interval (CI) 2.02, 2.56). Among males the prevalence was 5.25 % (95 %CI 4.46, 6.13) and among females was 1.35 % (95 %CI 1.13, 1.61) [6].

While reports suggest that most GW cases are asymptomatic, location, size, and number of the lesions usually determines the presence of symptoms, including pain, pruritus, and bleeding [7, 8]. While GW can be self-limiting, several patients require topical treatment or surgical procedures. The choice of treatment usually depends on the clinical assessment of the lesion, cost of the procedure, patients’ characteristics, and physicians’ preferences [9]. Although the treatment of GWs has been associated to increased individual and healthcare costs [10–12], only few countries in Latin America have studies assessing the costs and the economic burden of GW in the population [13–15]; Peru is not one of them.

Peru has a mixed healthcare system with public and private providers and insurers. While most of the private institutions are specialized in either service, public institutions are upstream integrated and therefore offer insurance and healthcare services [16]. The most important provider and insurer is the Ministry of Health (MoH) through the comprehensive health insurance (SIS by its acronym in Spanish – *Seguro Integral de Salud*) that covers 44.4 % of the population [17]. This structure has two implications. First, access to care its restricted by insurance membership; e.g., only holders of the SIS can be treated by the MoH. Second, most institutions are both a health services providers and payers. Therefore, the MoH, as the most important provider in the country, is expected to bear the biggest proportion of the costs associated to diagnose and treat GW in the country.

The MoH provides vaccination free of charge to all Peruvian citizens for all vaccines in the national immunization scheme. Peru introduced the bivalent HPV vaccine, that confers protection against the high-risk subtypes, 16 and 18 [18], for the first time in 2011 for girls from 9 to 14 years old. Just in 2016 the national vaccination scheme changed to the quadrivalent vaccine that also protects against the HPV-types 6 and 11 [19].

The objective of the study was to address the population-level costs of GW diagnosis and treatment, to present the economic burden of a disease, that could be prevented with a gender-neutral vaccine.

## Methods

### Study design

We conducted a cost-of-illness (COI) study aiming to estimate the total healthcare expenditures used to diagnose and treat people with GW. We used an activity-based (micro-costing) technique to estimate the cost of diagnosis and each treatment option from the provider’s perspective, the facilities under the purview of the Peruvian Ministry of Health (MoH).

The micro-costing technique decompounds each service (i.e., diagnosis and treatment options) into the inputs and quantity required to provide it. We then find the best price for each input and multiply it by the amount needed. The sum of all inputs provides an estimate of the cost per service. Since we used the provider’s perspective for the costing analysis, only direct medical costs (e.g. drugs, materials, equipment, and physicians’ and nurses’ wages) were included [20].

Since we used a prevalence-based approach, the COI is determined by the product of the prevalent cases and the average treatment cost [21, 22]. The prevalence was obtained from a previous study conducted by Garcia et al [6]. In the following sections we describe how each treatment’s technique cost was estimated, as well as how we arrived at the overall average treatment cost.

### Materials

We leverage the results found by Garcia et al [6] regarding the prevalence of GW, providers’ preferences for GW diagnostic methods, and distribution of cases across gender and type of case. Cases were categorized by physicians into “new” – no history of previous diagnosis, “resistant” – episode lasting longer than six months despite treatment, and “recurrent” – new case that appears within 12 months of previous episode. That study included physicians from six specialties: primary care physicians (including general practitioners and family medicine doctors), gynecologists, urologists, dermatologists, and infectious disease specialists.

**Fig. 1:**
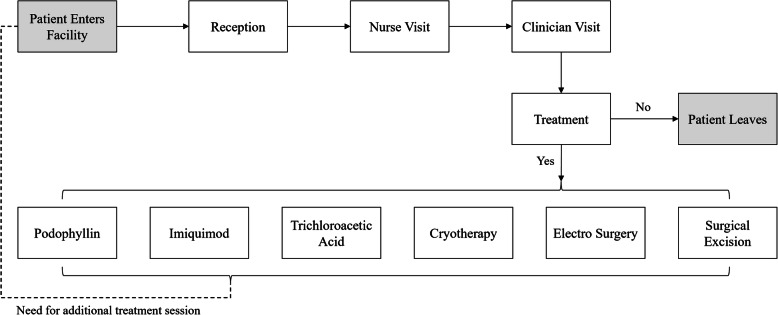
Clinical flow-map for genital warts treatment at a public healthcare facility in Peru

To identify resource use for a typical visit, we developed a flow map of key activities completed during a visit (Fig. 1). Then we conducted a review of national guidelines [23] to identify the materials used in each activity according to protocol. Additionally, we updated and improve this information with eight in-depth interviews with physicians that participated in Garcia’s study [6]. These interviews were used to get further information about treatment practices, preferences for specific treatments, materials and equipment used in each procedure, duration of each procedure, and validation of the treatment algorithms.

In 2016, we conducted primary costing-data collection in Lima (coastal city and capital of Peru), Ayacucho (Andean region), and Iquitos (Jungle). The selection of sites was purposive. Each site represents a major region in Peru and therefore it allowed us to collect the most heterogeneous costing data, resources utilization (i.e., quantity of the resources used), and clinical practices to create robust estimates. In addition, it is coherent with the study design of Garcia, et al., so it preserves internal consistency.

We interviewed a total of nine administrative and logistics officers that provided the unitary costs of all drugs, materials, medical supplies, and equipment used for each treatment option. From each interview we obtained purchasing data that contained, for each input, volume of purchase and price paid, or directly unitary cost. The unit cost of disposable inputs (e.g., cotton, needles) relies on the assumption that in every session the entire input is used (i.e., no partition for reuse). To estimate the unitary cost of durable inputs (e.g., equipment, medical instruments) we used the depreciation method [24]. From the interviews with administrative officers, we obtained the total cost of the good, enquire about the rotation period (e.g., how often an equipment is changed, or infrastructure renovated), from which we obtained the useful life, which we finally used to estimate the depreciation cost per minute. Thus, the unitary costs were in the same unit as the duration of each activity.

Regarding human resources, we used the opportunity cost of the paid-time of the healthcare workers(HCW) [25]. We obtained salary data from the National Registry of Healthcare Personnel (INFORHUS) from the MoH to improve the precision of the wage estimates. We used this information to estimate the cost per minute per type of HCW and the estimated time per activity reported by the interviewed physicians to calculate the attributable costs to each treatment. We obtained the costs for six key HCW: receptionist, file staff, cashier, nurse, physician, pharmacist, whose regular activities are fairly differentiated and therefore we minimized the risk of overlapping. We used the information from the validate flow map (Fig. 1) to match activities with the HCW that most likely will perform them.

From this information we estimated mean and standard deviation (SD) of the unitary cost of each input. In all cases outliers were excluded if a value was a at least 3 times bigger than its peers. While a few inputs have a large variability (see supplemental material sheet “Costing data”) we decided in favor of the mean, instead of quantile-based metrics such as the median, because it easier to communicate, it allows for a more straightforward implementation of the probability-based sensitivity analysis, and the impact of each individual input is too small to bias the results. The SD captures the variability of the unitary cost given regional differences, purchase preferences, and others.

### Analysis

First, we estimated the cost per session for a diagnosis appointment and each treatment technique. This is the sum-product of the resources’ amount needed to provide a service and its unitary costs. To account for the variability of the unitary costs we performed a Monte Carlo simulation [26]. We used a random number generator to obtain 1,000 estimates of each unitary cost based on a gamma distribution – the recommended distribution for cost data [27] – parameterized using the mean and standard deviation. Each draw from the distribution for all parameters is a simulation of the costing data, and therefore we obtained 1,000 simulations of the data. We estimated the final average costs 1,000 times and we were able to obtain the 2.5 % and 97.5 % percentiles of the distribution. Some parameters had no sample variability when only one source of information was obtained. In those cases, we assumed a SD of 1e-9 to conduct the simulation. Following the same process, we decomposed the cost per session into categories of costs: human resources, infrastructure, equipment, drugs, medical instruments, disposable materials, and public services. We present both the cost per session and the cost per cost category.

Second, in our study, the GW are compartmentalized by the combination of four groups given by the biological gender of the patient (2 categories), the type of case (3 categories), the physician’s specialty (5 categories), and treatment technique received (6 categories), resulting in 180 possible combinations. Each combination is called a compartment. The probability across compartments is not homogeneous and hence we sought to find the specific distribution of cases for each one. We used the information reported by Garcia et al [6] to estimate the distribution of cases given the combination of patient’s gender, type of case, and physicians’ specialty. The probability of no receiving treatment care once the warts have been detected vary across specialties but in all cases is negligible (see Garcia et al. [6]). The in-depth interviews provided us with the probability of choosing each treatment by physician specialty. We use these probabilities to estimate the final probability of each compartment. We report all the probabilities used for this analysis.

Third, we estimated the annual cost of treatment per patient as the product of (a) the cost of each session plus the cost of the diagnosis appointment, (b) the number of times the treatment was applied to each patient, dependent upon patients’ characteristics, type of case (new, recurrent, or resistant), and physicians’ preferences, and (c) the number of episodes within a year for recurrent cases. Thus, we obtained the annual cost per patient in each compartment. We report the average number of sessions per treatment, the overall number of episodes in recurrent cases by gender, and the final cost by treatment.

Fourth, we obtained an estimate of the average cost of diagnosing and treating a typical patient in one year as the sum-product of the annual cost per patient in each compartment and the associated compartment’s probability. Hence, this corresponds to a weighted average, where the weights are the probabilities of observing each combination of patient’ characteristics and physicians’ preferences. Given the distribution estimated for the cost per session (first point), we can estimate 95 % confidence interval for the annual cost of treating a typical case of GW.

Fifth, the COI of diagnosis and treating GW, is the product of the average treatment cost and the prevalence of GW. We calculate the point-estimate and range of feasible values of the COI. The point-estimate is the product of the mean values of prevalence and the average treatment costs. The lower bound is the product of the 95 %CI’s lower-bound for both the prevalence of genital warts and the cost of treatment; conversely, the upper-bound uses the upper-bound of the 95 %CI for both metrics.

Finally, the number of GW cases is based on the most recent estimation of population by age and gender, in 2017 the population of Peruvians between 18 and 60 years old was 18.4 million, 9.3 of them are males and 9.1 females [28].

All the costing data was collected in 2016 Peruvian Soles (PEN), but the results are expressed in 2019 US Dollars (USD) using a fixed conservative exchange rate of 3.3 PEN for each USD, and a yearly inflation of 2.5 %.

## Results

The most used techniques to treat GW are three topical (podophyllin, imiquimod topical, trichloroacetic acid – TCA) and three surgical (cryotherapy, electro surgery, and surgical excision). Table 1 shows the estimated cost for each treatment in a single session and the distribution among categories of resource input. Among treatment options, the most expensive was the surgical excision 24USD (95 %CI 12, 57), and the cheapest was TCA with a cost per session of 11USD (95 %CI 6, 18). For all treatment options, the category representing the largest share was human resources, whose cost per-session accounted for 70–95 %. The second most important category is disposable materials varying from 1 to 21 % of the total cost per session. This included all goods that are used just once and then disposed of. Supplemental Material contains all costing data used to make these estimations.

**Table 1:** Cost for each treatment in a single session, by categories of cost

	Diagnosis Appointment	Podophyllin	Imiquimod	TCA	Cryotherapy	Electro Surgery	Surgical Excision
**Cost per session (USD)**	**6.04 (2.5, 11.8)**	**12.08 (7.4, 19.3)**	**10.94 (6.2, 18.3)**	**10.84 (6.1, 18.2)**	**15.4 (8.1, 26.8)**	**17.44 (9.4, 29.3)**	**24.59 (12.3, 57.6)**
**Category of cost (USD)**							
Human Resources	6.03 (2.4, 11.8)	9.21 (4.5, 16.7)	9.21 (4.5, 16.7)	9.21 (4.5, 16.7)	13.39 (6.2, 24.7)	13.39 (6.2, 24.7)	13.86 (6.2, 26.4)
Infrastructure	0.08 (0.08, 0.08)	0.09 (0.09, 0.09)	0.09 (0.09, 0.09)	0.09 (0.09, 0.09)	0.28 (0.27, 0.29)	0.7 (0.69, 0.71)	0.44 (0.43, 0.44)
Equipment	-	-	-	-	0.52 (0.4, 0.7)	1.08 (0.01, 6.5)	-
Drugs	-	1.06 (1.06, 1.06)	1.44 (1.44, 1.44)	0.86 (0.86, 0.86)	-	-	0.96 (0.4, 1.86)
Medical instruments	-	-	-	-	-	0.19 (0.18, 0.19)	0.69 (0.68, 0.7)
Disposable Materials	0.05 (0, 0.25)	2.01 (1.91, 2.28)	0.46 (0.26, 0.79)	0.93 (0.73, 1.29)	1.47 (0.69, 4.82)	2.3 (0.46, 6.82)	9.23 (2.04, 40.58)
Water and Electricity	0.07 (0.07, 0.07)	0.08 (0.08, 0.08)	0.08 (0.08, 0.08)	0.08 (0.08, 0.08)	0.22 (0.22, 0.22)	0.32 (0.32, 0.32)	0.19 (0.19, 0.19)

USD: United States Dollars; TCA: trichloroacetic acid

The “cost per session” row represent the cost in which the payer incurs every time a physician applies a given treatment. Usually, each treatment is applied more than once depending on the physician’s assessment, who considers type of case (new, recurrent, or resistant), and treatment characteristics

Cells marked with “-“ indicates that the treatment did not employ those resources

Across all treatments, on average, a new case received 2.5 (min: 1, max: 4) sessions of treatment, while a resistant case received 3.4 (min: 1, max: 6). For recurrent cases, the average number of episodes in a year is 1.7 for males and 1.6 for females. Using this information, we estimated the annual average cost per treatment. The most expensive treatment was cryotherapy at 78USD per patient, followed by podophyllin at 58USD, and electrosurgery at 55USD. In contrast, surgical excision was the cheapest treatment at 36USD per patient. (Table 2) For detailed information on the distribution of probabilities across all compartments, please refer to Supplemental material.

**Table 2:** Average number of sessions, final cost, and probability of usage for each treatment

	Average Sessions per treatment			
	**New (Min, Max)**	**Resistant (Min, Max)**	**Annual average cost per treatment (USD)**	**Probability of treatment usage**
Podophyllin	3.2 (2.5, 3.7)	4.0 (4.0, 4.0)	58.6	39.6 %
Imiquimod	3.0 (2.5, 4.0)	2.7 (2.4, 3)	46.5	5.3 %
Trichloroacetic Acid	2.5 (1.6, 3.3)	5.3 (2.0, 8.0)	52.7	16.4 %
Cryotherapy	3.4 (2.8, 4.0)	5.1 (3.0, 6.2)	78.2	17.4 %
Electro Surgery	2.0 (1.1, 4.0)	2.1 (2.0, 2.3)	55.5	20.3 %
Surgical Excision	1.0 (1.0, 1.0)	1.0 (1.0, 1.0)	36.1	0.9 %

USD: United States Dollars

“Average sessions per treatment” indicates the number of sessions until episode resolution. “Probability of treatment usage” column shows the proportions of cases in which each treatment was used. “Annual average cost per treatment” includes cost of diagnosis and control sessions, information of number of sessions per treatment, and proportion of recurrent cases treated with each technique

This table presents summary data. Hence, the sum-product of the last two columns will differ from the reported annual average cost of treatment per person (59.9USD)

**Fig. 2:**
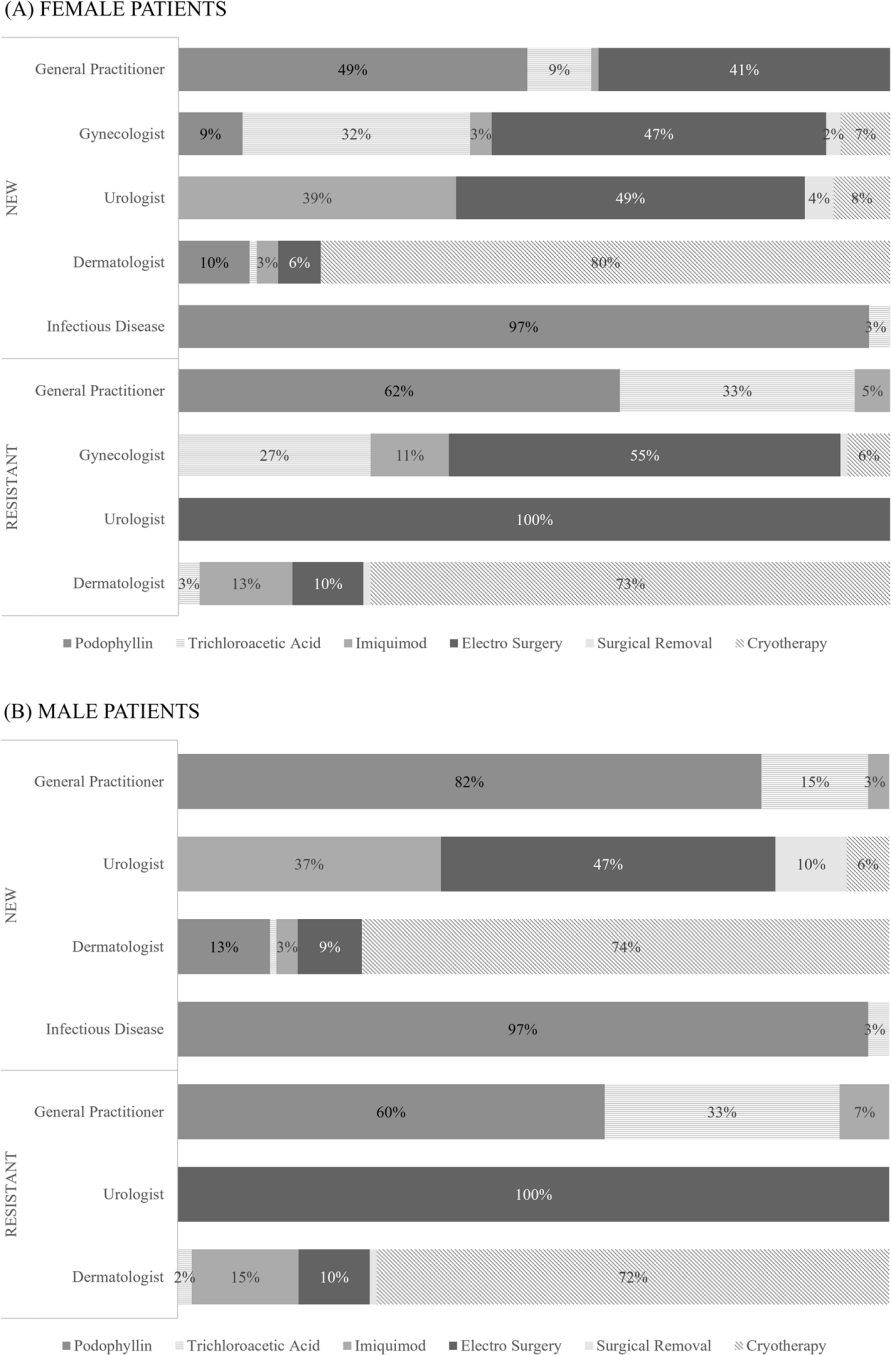
Distribution of treatment preferences by type of case, physicians’ specialty, and gender of the patient. Upper panel (**A**) corresponds to female patients, and lower panel (**B**), to female patients. Sections with no label represent 1 %

After estimating the probabilities for all possible combinations, podophyllin was the most frequently chosen treatment (40 %), followed by electrosurgery (20 %), cryotherapy (17 %), TCA (16 %), imiquimod (5 %), and finally surgical excision (1 %). (Table 2) Fig. 2 shows the distribution of treatment choice within physician specialty and type of case. While there is a lot of variability across physicians’ specialty, we observe some patterns. For instance, there was a preference for podophyllin by general practitioners which represents the biggest proportion of cases, and dermatologists preferred cryotherapy in any circumstance.

We estimated the average treatment cost in 59.9USD. From the Monte Carlo simulation we estimate the 95 % credibility interval in 45.5 to 77.6USD. The distribution of the simulations behaves as normal with skewness 0.3 and Kurtosis 3.1. Considering the distribution of cases across gender in the sample, we estimated the annual average cost of treating a male in 61.3USD and a female in 58.9USD.

Given the size of the population of the prevalence, we estimate 333,709 annual cases of GW among males and 85,811 among females. Thus, the estimated COI is 25.1 million USD, with a feasible range of 16.9 to 36.5 million USD. Given that the estimated prevalence of GW in males is 3.9 higher than in females [6]. Considering this, the estimated COI for females is 5.1USD million, while the estimated COI for males is 20 million.

## Discussion

This is the first study to estimate the economic burden of treating GW in Peru and one the few in Latin America. Our study leverages information from multiple sources to estimate the cost of several techniques to treat GW, and the associated annual COI for Peruvian healthcare sector. Our statistical approach explicitly incorporates the key factors that determined which treatments are used under what conditions via the assessment of usage probabilities, as well as the uncertainty of the costing data.

We found that human resources account for 70–95 % of the cost per treatment. While these values might look surprising, they are a consequence of the use of diagnosis and treatment techniques that are more intensive in time from healthcare workers than in equipment or other resources. In the selection and implementation of the methods for this study, we accounted for the known issues related to attribution of shared costs [29], that can lead, among others, to overestimation of human resources. We followed several procedures to ensure an accurate estimation of costs. First, the attribution of healthcare worker by activity was based on a validated flow map. Second, we selected healthcare workers whose activities would less likely overlap to prevent mismatching. Third, the costs estimation was based on governmental data and cleaned from outliers.

Our estimation of 25.1USD million for the COI of GW represents the total expenditures that the Peruvian MoH would face in diagnoses and treatment of GW in a year. There are few caveats that are important to highlight regarding this estimation. First, the prevalence used was based on formal care; it does not include self-treated cases and the cost in which these patients incur are not considered in our estimation of the COI.

Second, per the structure of the Peruvian heath system, only holders of the SIS are eligible to be treated by the MoH [16]. An important proportion of people with no insurance would seek and receive treatment in the MoH because it is cheaper than other providers. However, they would need to pay out-of-pocket for all services received. Given the demand size and associated negotiation power, the MoH prices are lower than other providers, making our COI estimate rather conservative of the total cost of treating GW in the public sector. On the other hand, stock-outs in MoH operated-facilities and other conditions have been associated with out-of-pocket expenditures in SIS-holders [30] which creates an opposite effect over the COI as some of the MoH costs could be transferred to individuals.

Third, our analysis implicitly assumes that all patients with detected GW would receive one of the six studied treatment options. According to Garcia et al [6], the proportion of cases that are left untreated are 0.16 % for females and 0.17 % for males. Considering the small number of cases that do not receive treatment once are detected, we did not impose any correction on the prevalence to estimate the COI. On the other hand, in both, Garcia et al. study and our in-depth interviews, the six treatments we analyzed here were the most frequently used ones. Although other treatments were reported, such as interferon or fluorouracil, the proportion of usage was very small and out of the scope of our priorities. We do not have evidence that the inclusion of these treatments would importantly change our results, especially considering the small variability across the cost per session of topical treatments.

Despite these caveats, the estimated COI represent an important amount of money. According to the Peruvian Ministry of Economy and Finance [31], in 2019 the budget for individual health, which includes all actions that aim to treat and rehabilitate people, was around 5.2 billion USD (15.6 billion PEN; the total budget for the MoH was 20.9 billion PEN), equivalent to 145 USD per capita. Thus, the cost to diagnose and treat GW, as conservative as it is and without considering out-of-pocket expenditures, represents 0.16 % of the total institutional budget. Further, the average per person cost of GW, represents over a third of the per capita value destined to individual health.

Few studies in Latin America have estimated the cost of diagnosis and treatment of GW. For ease of comparison across countries we converted our estimates into international dollars (intl.D) using the purchase parity pawer (PPP) factor published by the World Bank [32]. Considering a PPP factor of 1.74, we estimate the annual average cost of treatment per person in 104.2 intl.D (95 %CI 79.2, 135), and the COI in 43.7intl.D million (uncertainty interval 29.4, 63.5).

An Ecuadorian study used a societal perspective using expert consultation to determine clinical practices. They found that the average cost of treatment varies from 205.4 to 251.7intl.D (PPP factor 0.52), depending on the treatment used [14]. Our results cannot be compared to these results because the authors used a societal perspective, which includes a broader range of costs than to our analysis and includes private practices. Our study was based on the payer’s perspective and only from public facilities which tend to be cheaper than private options.

In Mexico, the average cost for diagnosis and treatment of GW was found to be 1,326.8 1,418.5intl.D (PPP factor of 9.15) for men and women, respectively [15]. This study used the healthcare system perspective, and the information was obtained through specialists’ interviews. Our estimates are lower than these results, due to the cost of a diagnosis. According to Garcia et al [6]. the diagnosis in Peruvian facilities is made using visual inspection in more than 95 % of the cases; a very cheap technique for which the diagnosis visit is 10.4intl.D. In the Mexican study, consulted physicians reported using laboratory test to diagnose GW. Hence, the average diagnosis appointment alone was 803.4intl.D.

GW is a vaccine-preventable disease and therefore the costs associated to its diagnosis and treatment can be mitigated [33, 34]. In the United States, quadrivalent vaccine was introduced for females in 2006, and subsequently to males in 2009 [35, 36]. Although the vaccine achieves its highest efficacy in HPV non-exposed individuals (i.e. before sexual initiation, usually younger than 13 years old), the vaccine has been recommended for everybody up to 26 years of age [37]. As a result, there has been a reduction of 0.8 % of genital warts between 2007 and 2014 [38, 39]. Australia is probably an even better example. The country was the first to introduce the quadrivalent HPV vaccine implemented through a national program, targeting girls aged 12 and 13 years, with an additional two catch-up campaigns from 2007 to 2009 targeting women up to 26 years old [40]. In 4 years, the proportion of women under 21 years old diagnosed with GW was reduced from 11.5 % to 2007 to 0.85 % in 2011. Similar declines were observed in men under 21 years of age and men and women of 21 to 30 years old [41, 42].

Under the current immunization scheme, only girls are eligible for the quadrivalent HPV vaccine [19]. However, there is evidence that vaccinating boys could also be beneficial at the individual level, by reducing incidence of associated cancers and GW, and at the community level, by reducing the spread of the HPV [43, 44]. Considering the high concentration of GW cases in males [6] and the estimated higher cost of providing them care, a gender-neutral HPV vaccination approach could potentially save millions of dollars by preventing GW in Peru and improve health outcomes for both males and females [45]. Further, according to the pricelist of the Pan American Health Association (PAHO) the cost for the two-dose quadrivalent HPV-vaccine is 19.9 USD, almost a third of the average cost of treating a GW. Although a study would need to determine how many GW infections the quadrivalent vaccine can prevent in Peru under specific uptake scenarios and target population, two studies in Latin America have found that it can significantly reduce the incidence of GW in up to 80 % [46, 47].

This study was not without limitations. First, we did not collect costs for other healthcare providers besides the MoH and therefore the estimated COI is a conservative number compared to the *true* cost of treating GW bore by the Peruvian healthcare system. Second, this study uses the prevalence values and physicians’ reported preferences for treatment presented by Garcia et al [6] and hence subject to the same limitations and potential sources of bias as presented in that study. Third, the probabilities used to estimate the distribution of cases across combinations of patients’ and physicians’ characteristics were obtained through interviews and hence subject to recollection and social-desirability biases. Nonetheless, the application of our instruments followed best-practices for data collection [48] and we are confident on the accuracy of our results within its limitations.

## Conclusions

To our knowledge, this is the first study that analyzes the economic burden of GW in Peru, and one of the few in Latin America. We used a micro-costing technique with data collection in multiple settings to account for the regional price variability. Our fieldwork collection was supplemented with governmental data, which improve the precision of our estimates. Finally, we explicitly assess uncertainty in our estimates by including confidence intervals and performing a Monte Carlo simulation to estimate credible intervals for the average cost of treatment.

We hope our findings bring attention to the economic consequences of diagnosing and treating GW, and the burden that it represents to the MoH and the Peruvian Healthcare sector at large and becomes another reason to make the decision of moving forward into gender-neutral vaccination.

## Supplementary information

**Additional file 1**

## Data Availability

The authors have made available all data and materials in the Supplemental file.
